# Ontogenetic Scaling of Fore- and Hind Limb Posture in Wild Chacma Baboons (*Papio hamadryas ursinus*)

**DOI:** 10.1371/journal.pone.0071020

**Published:** 2013-07-29

**Authors:** Biren A. Patel, Angela M. Horner, Nathan E. Thompson, Louise Barrett, S. Peter Henzi

**Affiliations:** 1 Department of Cell and Neurobiology, Keck School of Medicine, University of Southern California, Los Angeles, California, United States of America; 2 Department of Ecology and Evolutionary Biology, Brown University, Providence, Rhode Island, United States of America; 3 Department of Anatomical Sciences, Stony Brook University, Stony Brook, New York, United States of America; 4 Psychology Department, University of Lethbridge, Lethbridge, Alberta, Canada; 5 Applied Behavioural Ecology and Ecosystem Research Unit, University of South Africa, Florida, South Africa; Midwestern University & Arizona State University, United States of America

## Abstract

Large-scale interspecific studies of mammals ranging between 0.04–280 kg have shown that larger animals walk with more extended limb joints. Within a taxon or clade, however, the relationship between body size and joint posture is less straightforward. Factors that may affect the lack of congruence between broad and narrow phylogenetic analyses of limb kinematics include limited sampling of (1) ranges of body size, and/or (2) numbers of individuals. Unfortunately, both issues are inherent in laboratory-based or zoo locomotion research. In this study, we examined the relationship between body mass and elbow and knee joint angles (our proxies of fore- and hind limb posture, respectively) in a cross-sectional ontogenetic sample of wild chacma baboons (Papio hamadryas ursinus) habituated in the De Hoop Nature Reserve, South Africa. Videos were obtained from 33 individuals of known age (12 to ≥108 months) and body mass (2–29.5 kg) during walking trials. Results show that older, heavier baboons walk with significantly more extended knee joints but not elbow joints. This pattern is consistent when examining only males, but not within the female sample. Heavier, older baboons also display significantly less variation in their hind limb posture compared to lighter, young animals. Thus, within this ontogenetic sample of a single primate species spanning an order of magnitude in body mass, hind limb posture exhibited a postural scaling phenomenon while the forelimbs did not. These findings may further help explain 1) why younger mammals (including baboons) tend to have relatively stronger bones than adults, and 2) why humeri appear relatively weaker than femora (in at least baboons). Finally, this study demonstrates how field-acquired kinematics can help answer fundamental biomechanical questions usually addressed only in animal gait laboratories.

## Introduction

For centuries, biologists have observed that small mammals tend to have crouched limb postures with flexed joints, whereas larger animals move with erect limbs and extended joints (e.g. [1]–[4]). Biomechanical models suggest that crouched postures require greater muscle force to counteract torques generated by substrate reaction forces (typically at midstance when forces are highest), contributing in turn to high bending strains in long bones [4]. In contrast, by increasing the effective mechanical advantage (EMA; ratio of muscle moment arm to substrate reaction force moment arm) of the anti-gravity muscles, erect postures help attenuate the magnitude of joint moments [4], thereby reducing compensatory muscles forces and potentially moderating bone strain. Additional attenuation of bone strain can ­be obtained by aligning limb segments more closely with the resultant of the substrate reaction force vector during weight support, which also minimizes shaft bending moments [4]–[6]. By using erect limb postures, larger animals are able to maintain tissue mechanical safety factors [bone: between 2 and 4] similar to those of smaller animals without ‘over-building’ the skeletal system [6]–[8].

The best evidence supporting this relationship between limb posture and body size comes from a large-scale interspecific study of kinematic data from mammals spanning 0.04 to 280 kg in size, in which limb EMA correlated positively with body size [4]. Analyses of the scaling of limb posture within narrower phylogenetic ranges (i.e., clade specific), however, have yielded mixed results. Some studies, such as one on rodents [7] and one on cercopithecoid primates [9], found similar trends of increasing joint angle with body size but with weaker statistical support. There is, however, more convincing support from morphological indicators of limb posture in a broad sample of primates (e.g., mid-shaft cross-sectional geometry of the femur [10] and subchondral bone radiodensity patterns in the distal femur [11]). Conversely, a kinematic study on felids only found a correlation between body size and one of the 12 measured limb posture characteristics (elbow angle at mid-stance), prompting the authors to suggest that there is no scaling phenomenon between body size and limb posture in this group [12].

Several factors likely contribute to the lack of congruence among these analyses. A primary and acknowledged concern among all of the previously cited studies is sampling bias, both within and across species. For example, nine of the 14 species used in Biewener's [4] original study were rodents, and the largest rodents (capybaras) are a semi-aquatic species [13], and thus may deviate from the generalized rodent pattern. Similarly, Polk [9] was only able to include two each (one male and one female) for three species of cercopithecoid primates yielding a total sample size of six individuals. Although Day and Jayne [12] were able to examine nine species of felids that ranged in mass from ∼3.7 kg to 200 kg, the number of individuals per species was small (N<6) and consequently data from both sexes were pooled. This could be problematic as felids are sexually size dimorphic, with male tending to be much larger than females [13]. Furthermore, previous studies have shown that pooling of sexes can confound kinematic studies since males and females often display sex specific biases in body size, differ in skeletal shape, and often differ in locomotor and positional behavior (see examples in [14], [15]). Finally, these data were collected in laboratory settings or unnatural outdoor enclosures (e.g., zoos), environment that may affect the postural and locomotor kinematics of animals (e.g. [16], [17]).

An alternative approach that avoids the confounding influence of different phylogenetic histories is to focus on limb postural change as a function of ontogeny within a taxon (e.g. [18], [19]). The range of body sizes a single species experiences during growth can span several orders of magnitude, particularly in altricial mammals such as primates [20]. For example, in Vilensky and Gankiewicz's [21] ontogenetic study spanning a three-fold mass range in vervet monkeys, some individuals were observed to have more extended knee joints when they were heavier and older compared to when they were younger and lighter. However, Young [19], [22] did not observe statistically significant differences in knee joint angles in older, heavier squirrel monkeys compared to younger, lighter individuals (0.2–0.5 kg size range) even though the larger-bodied individuals did experience greater hind limb forces relative to forelimb forces. Potential sources of error in these studies again include small sample size (five individuals were used in each study), as well as locomotion behavior altered by treadmill use (in the case of the vervet monkeys), and general kinematic differences due to the laboratory setting [17]. It also may be the case that the animal models used were not large enough to elicit change in limb posture. Small animals (typically less than <1–2 kg) are able to limit bone stresses while in crouched postures (e.g. [5]) and limb posture of small species is likely determined by behavioral influences (e.g., crypsis, maneuverability) rather than by biomechanical constraints (e.g. [5], [11]).

In an effort to both take advantage of the insights offered by body size change over ontogeny and overcome the sample size limitations of lab based studies, we examine scaling of limb posture in a cross-sectional ontogenetic sample using a field-based approach. We selected a wild troop of chacma baboons (*Papio hamadryas ursinus*) that are well-suited for this line of inquiry because they (1) live in large multi-male/multi-female groups with typically more than 35 individuals [23], (2) undergo >10 fold increase in body size between birth and adulthood (see Table 1), and exhibit large size differences between sexes (where adult males can outweigh adult females by ∼15 kgs [24]), and (3) are (semi-) terrestrial and can easily be viewed where they occupy and utilize open habitats [23]. Moreover, infant baboons are able to locomote independently at an early age (2–5 months [25]), and previous ontogenetic studies of a closely related baboon subspecies (*Papio hamadryus cyncocephalus*) in captivity demonstrated both morphological (i.e., posterior center of mass shift) and kinematic (e.g., stride and step length) changes between infants and young juveniles [15]. Similar morphological, behavioral and biomechanical changes likely take place in wild baboons. Finally, Polk [26] reported that of the three cercopithecoid species in his study sample, the baboons showed the strongest support for Biewener's [4] biomechanical model. Thus, if body size is a primary determinant of limb posture, then we predict that older, larger individuals will walk with more extended fore- and hind limbs compared to younger, smaller individuals. Additionally, we predict that smaller juveniles will walk with more variation in limb posture than large adults.

**Table 1:** Tables 1. Sample and descriptive statistics.

Individual	Sex	Age (months)	Mass (kg)	Knee Joint Angle (degrees)				Elbow Joint Angle (degrees)			
				N	Mean	St. Dev.	CV	N	Mean	St. Dev.	CV
Sylvestor	Male	12.0	2.0	2	127	0.69	–	2	158	7.63	–
Oscar	Male	13.0	2.5	7	131	12.14	9.241	9	151	8.52	5.637
Chester	Male	13.0	3.3	1	127	–	–	1	157	–	–
Elissa	Female	20.0	4.0	3	136	4.06	–	3	161	4.15	–
Luke	Male	19.0	4.9	7	139	7.70	5.544	7	152	4.67	3.069
Emilio	Male	20.0	7.0	9	140	7.52	5.374	9	152	3.55	2.329
Bono	Male	36.0	7.0	4	147	1.43	–	6	158	4.01	2.533
Quincy	Male	46.0	9.0	14	134	4.80	3.592	17	156	4.22	2.710
Doug	Male	49.0	9.0	5	138	4.74	3.425	5	148	3.14	2.127
Turtle	Male	44.0	9.3	15	139	4.98	3.584	19	158	4.67	2.955
Kyle	Male	55.0	11.5	1	131	–	–	1	158	–	–
Cartman	Male	56.0	12.0	4	135	7.82	–	4	154	3.82	–
Vicky	Female	61.0	13.0	5	141	4.34	3.066	5	156	4.05	2.595
Ulrike	Female	62.0	13.0	4	136	7.19	–	5	152	2.41	1.590
Kevin	Male	62.0	13.3	6	135	3.52	2.612	7	147	4.46	3.026
Catherine	Female	–	16.2	0	–	–	–	5	159	4.18	2.624
Lynn	Female	–	17.0	7	132	4.16	3.145	6	157	1.30	0.829
Rushenka	Female	87.0	17.5	3	132	7.98	–	4	156	2.78	–
Jane	Female	–	17.5	8	140	3.89	2.777	6	155	5.01	3.237
Alice	Female	–	17.8	9	139	4.14	2.990	8	154	5.40	3.508
Olga	Female	–	17.8	9	134	4.72	3.510	9	157	4.27	2.725
Alison	Female	–	18.0	3	142	1.44	–	3	157	3.74	–
Emma	Female	–	18.0	1	134	–	–	1	151	–	–
Sarah	Female	–	18.5	8	140	5.18	3.704	9	161	4.09	2.538
Christina	Female	–	19.0	5	141	1.30	0.920	5	160	8.03	5.019
Watson	Male	108.0	24.0	22	145	4.36	3.012	26	160	4.34	2.713
Guy	Male	–	26.0	3	143	1.35	–	3	162	4.27	–
Pinker	Male	–	26.0	6	144	1.47	1.017	7	154	5.38	3.490
Schwartze	Male	–	27.0	12	140	3.10	2.211	10	161	4.43	2.751
Caliban	Male	–	28.0	4	137	5.59	–	4	155	4.64	–
Prof Higgins	Male	–	28.0	10	144	5.23	3.627	11	158	4.13	2.621
Redfur	Male	–	29.0	16	139	4.82	3.476	12	156	4.93	3.150
Seth	Male	–	29.5	8	149	2.15	1.440	9	159	2.65	1.667

## Materials and Methods

**Figure 1:**
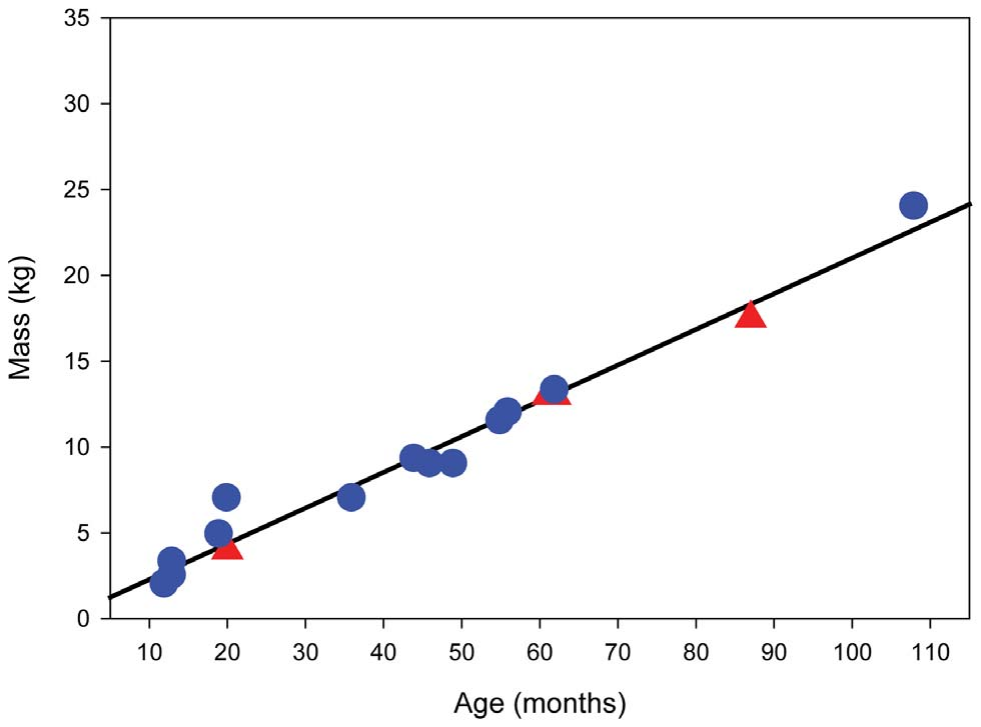
Age vs. mass. Correlation between known age (in months) and body mass (in kg) for a subset of the comparative sample. Red triangles are for females. Blue circles are for males. Statistics: r^2^ = 0.974; *p*<0.001.

The study site was the De Hoop Nature Preserve (34°27′00′′S, 20°24′00”E) in the Western Cape Province in South Africa. De Hoop is a winter rainfall habitat characterized by endemic fynbos habitat that is home to a large population of chacma baboons. The chacma baboons are not a protected species in De Hoop Nature Preserve, and no permits were necessary for this study. Data come from a single, habituated troop (VT) that has been studied since 1997 [27]. Videos of all animals used in this study were recorded in July 2004 when the troop was making extensive use of the area adjacent to a large inland lake. The open, flat terrain facilitated collection of locomotion data from a large number of individually identifiable animals of all age-sex classes. The focal animals (total N = 33) included both males (N = 20) and females (N = 13) with an age range of 12 to >108 months and a body mass range of 2 kg to 29.5 kg (Table 1). A custom built, portable electronic digital scale placed opportunistically at sleeping sites was used to obtain body mass data. We found that age (in months) and body mass (in kg) were significantly correlated (r^2^ = 0.974; *p*<0.001; Fig. 1). A Shapiro-Wilk test for normality revealed that the body mass data in our sample follow a normal distribution (*p* = 0.103) and therefore we did not log transform this variable in our statistical analyses (see below). It is necessary to point out that although we had a diverse sampling of males across all body size ranges, most of our female sample comes from older, larger individuals. All juveniles and sub-adults were already weaned and were independent of their mothers for locomotor behaviors.

**Figure 2:**
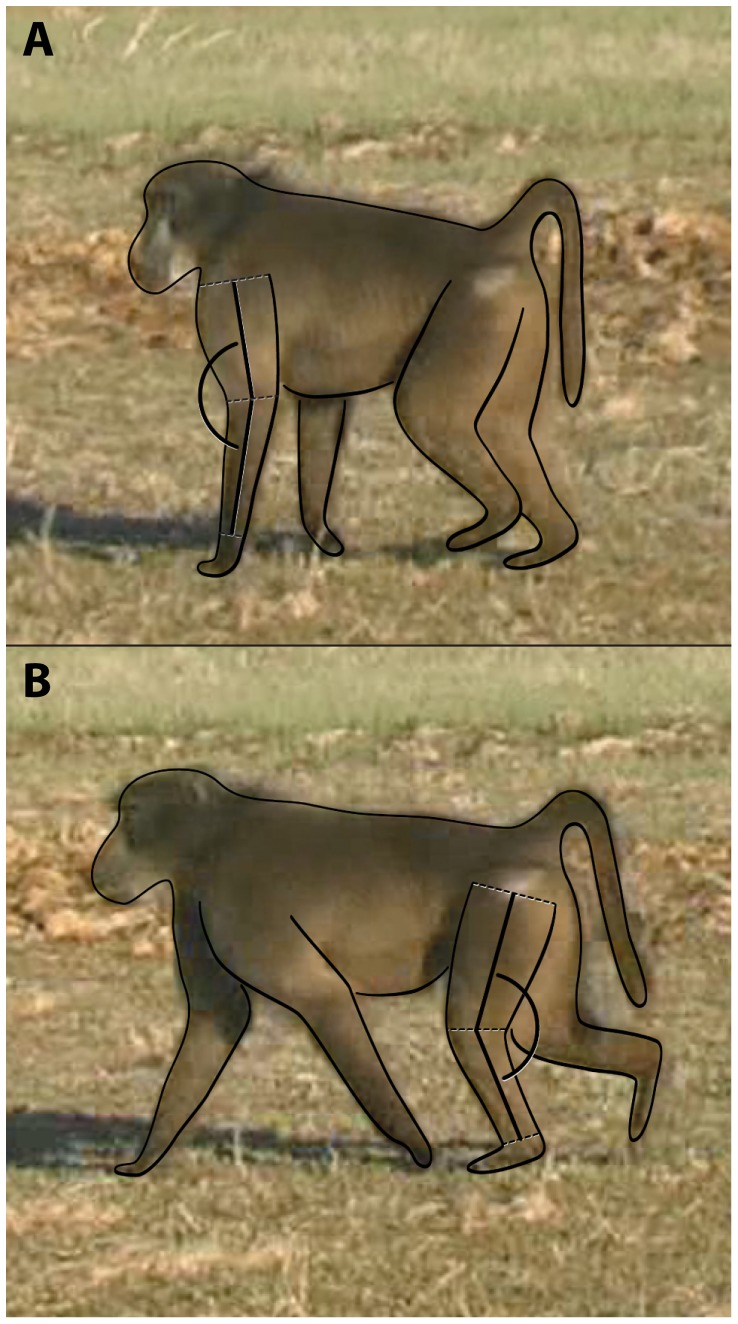
Angle measurements. Illustration showing how joint angles (in degrees) were measured. (A) Elbow joint angle was measured as the angle between the arm segment and the forearm segment. A line determined the arm segment with its proximal end approximating the midpoint between the anterior and posterior contours of the arm at the shoulder joint and the distal end approximating the midpoint between the anterior and posterior contours of the elbow joint. A line determined the forearm segment with its proximal end approximating the midpoint between anterior and posterior contours of the elbow joint and the distal end approximating the midpoint between the anterior and posterior contours of the wrist joint. (B) Knee joint angle was measured as the angle between the thigh segment and the leg segment. A line determined the thigh segment with its proximal end approximating the midpoint between the anterior and posterior contours of the thigh at the hip joint and the distal end approximating the midpoint between the anterior and posterior contours of the knee joint. A line determined the leg segment with its proximal end approximating the midpoint between anterior and posterior contours of the knee joint and the distal end approximating the midpoint between the anterior and posterior contours of the ankle joint.

Video was recorded (60 frames/s) with a commercial handheld digital video camera placed on a tripod. The camera was typically positioned in close proximity to the focal animal (∼2–3 meters). Trials were included in the analysis only if the animal appeared to be moving in a straight line and perpendicular (<20 degrees relative) to the camera for two consecutive strides while on the ground [28]. Ultimately, sample sizes ranged between 1–26 trials per individual (Table 1). Angle measurements were obtained from still-frame images of each video sequence during fore- and hind limb mid-support, similar to previous published studies of primate kinematics [29]–[31]. Mid-support was defined as the kinematic event when the wrist joint was below the shoulder joint and the ankle joint was below the hip joint. Mid-support is typically the time during the step cycle when the vertical component of the GRF vector is at its peak in baboons [32], thereby making it the most relevant kinematic event to address our hypotheses about limb loading. The low, scrubby vegetation of this field site facilitated viewing of the animal's hands and feet. Frame-by-frame analysis of video trials was performed using Virtual Dub software (http://www.virtualdub.org). Angle values (in degrees) from each trial were measured either using ImageJ software (http://rsbweb.nih.gov/ij/) or Didge software (http://biology.creighton.edu/faculty/cullum/Didge/index.html). Specifically, two-dimensional knee and elbow angles were measured between the thigh/leg and arm/forearm segments, respectively (Fig. 2). Each trial was measured at least three times and the average value was used in all analyses. Trials for each joint were measured by only one author (knee: AMH; elbow: NET). Joint angle values were considered more extended if they converged on 180 degrees. For subsequent statistical analyses, we calculated the mean joint angle (for all individuals) and the coefficient of variation (CV) about the mean value (for individuals that had five or more trials).

Because speed and gait can affect limb joint kinematics in baboons [26], [33], we chose trials only when duty factor was greater than 0.50 (i.e., kinematic symmetrical walks [34]). Duty factor was determined by taking the percentage of time the hind limb was in contact with the ground (i.e., step duration) relative to total hind limb stride duration. Ultimately, we found that duty factor was not significantly correlated with joint angles in any of the individuals (*p*>0.05) and thus all statistical analyses were performed without duty factor as a covariate.

Our analyses consisted of a series of Pearson's product moment correlations and least squares (LS) regressions. We regressed mean knee and elbow angles against body mass for each individual in the entire sample, and then again for each sex independently. Second, we regressed the CVs of knee and elbow angles against body mass in the entire sample and within each sex. Following Day and Jayne [12], we focused our attention on the correlation coefficient and the sign of the calculated slope (positive or negative). We were also interested in evaluating whether males adopt more extended fore- and hind limbs compared to females across all size ranges. Therefore, we performed a pair of analyses of covariance (ANCOVA) with body mass as the covariate. In the ANCOVA, least-square (LS) means for elbow and knee joint angle for each sex were calculated and then compared using a Tukey's HSD tests. All statistical analyses were performed in JMP v.9.0 (SAS Institute Inc.) or SPSS v.16 (SPSS Inc.) software packages.

## Results

**Figure 3:**
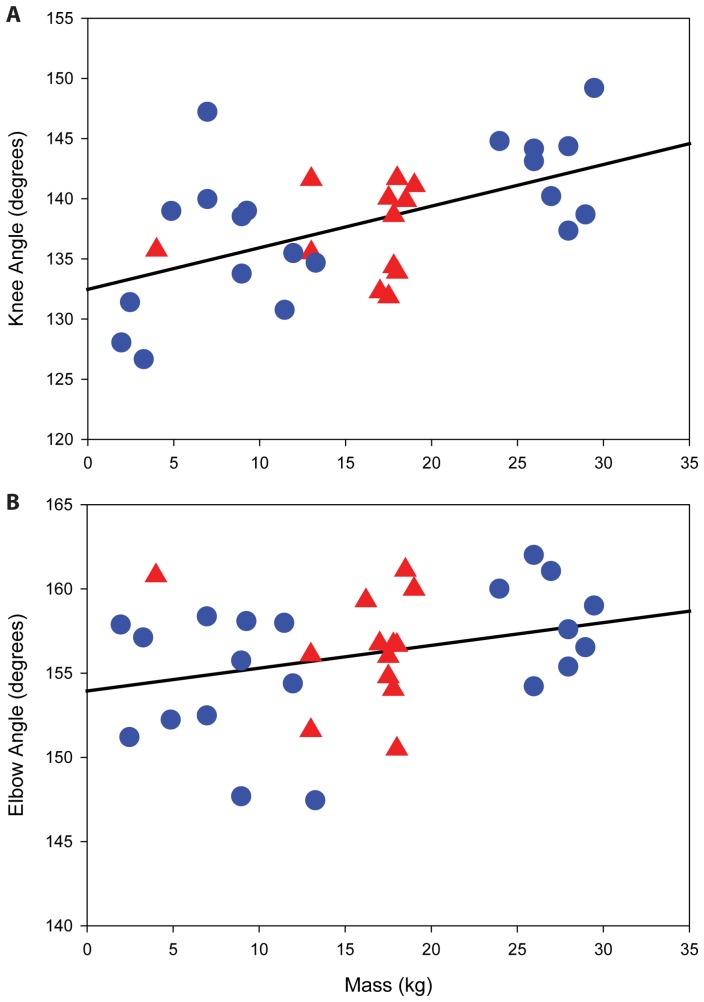
Body mass vs. mean joint angle. Relationship between mean knee joint angle (A), and mean elbow joint angle (B) and individual body mass. Data fit with a least squares regression line. Red triangles are for females. Blue circles are for males. See Table 2 for relevant statistics.

Descriptive statistics for knee and elbow angle variables are shown in Table 1. Results of regression analyses are presented in Table 2 and illustrated in Figure 3. For the entire sample, there was a positive significant relationship between body mass and knee angle (r^2^ = 0.301, *p* = 0.001). Although body mass had a small positive effect on elbow angle, this relationship was not statistically significant (r^2^ = 0.093, *p* = 0.084). Thus, as individuals mature and increase mass, only hind limbs become significantly more extended (Fig. 3).

**Table 2:** Results of least squares regressions against body mass*.

Variable	Sample	N	Slope	Intercept	r^2^	*p*
Knee Joint Angle	All individuals	32	0.346	132.47	0.301	0.001
	Males	20	0.372	132.51	0.386	0.004
	Females	12	0.096	135.68	0.012	0.732
Elbow Joint Angle	All individuals	33	0.135	153.94	0.093	0.084
	Males	20	0.163	153.27	0.176	0.066
	Females	13	−0.161	159.05	0.039	0.516
Knee Joint CV	All individuals	20	−0.144	5.85	0.448	0.001
	Males	13	−0.143	6.11	0.502	0.007
	Females	7	−0.123	4.99	0.070	0.566
Elbow Joint CV	All individuals	23	−0.019	3.17	0.026	0.465
	Males	14	−0.027	3.35	0.098	0.275
	Females	9	0.264	−1.66	0.248	0.173

* Significant at p<0.05.

When regressions between body mass and joint angle were performed within each sex separately, we found that the significant positive relationship between body mass and knee angle was upheld in the male-only sample (r^2^ = 0.386, *p* = 0.004), and that the slope and intercept of this relationship were similar to that of the regression equation for the combined sample (slope = 0.372 males only; slope = 0.346 both sexes). The female-only regression demonstrated no relationship between knee angle and body mass (r^2^ = 0.012, *p* = 0.732). Neither males nor females displayed a significant relationship between elbow joint angle and body mass, though males in the sample had a positive trend of body mass on elbow angle (r^2^ = 0.176, *p* = 0.066).

Although some of the relationships discussed above are statistically significant, their correlation coefficients can be considered rather low. After an outlier-analysis was performed on the spread of values in Figure 3 and Table 2, it became apparent that Bono, who is one of the smallest individuals at 7 kg, adopted a highly extended knee joint similar to Seth, who is the largest animal at 29.5 kg (147 vs. 149 degrees, respectively). When removing Bono from the analyses, r^2^ values increase and the *p* values decrease for the knee joint (combined sample: r^2^ = 0.424, *p*<0.001; male-only sample: r^2^ = 0.551, *p* = 0.0003).

For the combined sample, and in the male-only subset of the data, there was a significant negative relationship between body mass and knee joint CV (combined sample: r^2^ = 0.448, *p* = 0.001; male sample: r^2^ = 0.502, *p* = 0.007). Thus smaller individuals had more variation in hind limb posture. This relationship was not significant for the elbow joint in the combined sample and in the male-only sample (combined sample: r^2^ = 0.026, *p* = 0.465; male-only sample: r^2^ = 0.098, *p* = 0.275). For both the knee and elbow joints, body mass had no affect on limb posture variation in females (*p*>0.05; see Table 2).

Table 3 presents the distribution of LS means of knee and elbow angles for each sex that were calculated in the ANCOVA (with body mass as the covariate). Even though the LS means for knee angle appear larger in males compared to females, and the LS means for elbow angle appear larger in females compared to males, the Tukey's HSD tests demonstrate that males and females do not significantly differ from each other in both elbow and knee joint angles (knee joint: *p* = 0.473; elbow joint: *p* = 0.626).

**Table 3:** Least squares (LS) means and results of ANCOVAs between males and females*.

Variable	Sex	Mean (degrees)	LS Mean	Std. Error	*p*
Knee Joint Angle	Female	137	137	1.323	0.473
	Male	138	138	1.025	
Elbow Joint Angle	Female	156	156	1.006	0.626
	Male	155	155	0.811	

* Significant at p<0.05.

## Discussion

Large-scale interspecific analyses have previously demonstrated that larger species tend to walk with more extended limb postures. Such kinematic modifications can help decrease the magnitude of muscle force needed to counteract gravity, which in turn attenuates musculoskeletal stresses arising from both muscle and substrate reaction forces [4]–[6]. Some studies using more narrow phylogenetic samples have found support for this hypothesis (e.g., primates [9], [11]; rodents [7]), while others have demonstrated little to no relationship between body mass and limb posture (i.e., felids [12]). The goal of the present study was to test if large-scale interspecific patterns of limb orientation hold true in a more phylogenetically restricted but ontogenetically expanded sample, specifically in a population of wild chacma baboons (*Papio hamadryas ursinus*). By filming naturalistic locomotion in the wild, we were able to include a greater number of individuals spanning a large range of body size, as well as avoid many of the potential problems of studying posture and behavior inherent in laboratory studies. Furthermore, we were able to analyze males and females separately, which to our knowledge has not been rigorously investigated in previous studies. Using this approach, our findings for hind limb posture support Biewener's [4] biomechanical model in that older, heavier baboons tend to walk with more extended knee joints. In contrast to the model, however, elbow joint angles did not become more extended with an increase in body size.

The fact that knee angle, but not elbow angle, was positively correlated with body mass in our primate sample indicate that there may be different biomechanical demands imposed on hind limbs versus forelimbs over ontogeny in baboons. In general, primates tend to support a greater proportion of their mass on their hind limbs than on their forelimbs [32], [35]–[37] and thus a more extended hind limb could be a way to mitigate potentially higher musculoskeletal stresses acting on the thigh and leg bones. Baboons, however, are among the few primate quadrupeds that have an approximately equal distribution of mass on their fore- and hind limbs, particularly during ground locomotion [26], [32], [35], [38] making this an unlikely explanation for the different scaling relationships for elbow angle and knee angles in our wild chacma baboon sample.

In addition to not changing with increasing body size, the elbow joints of chacma baboons are more extended than the knee joint across ontogeny (as also seen in many other cercopithecoid monkeys in general; see [31]), which likely serves to create a longer effective forelimb length. Longer effective limb length can help to lower energetic costs associated with long distance travel [39], [40]. This could be important for terrestrial primates like baboons who spend a significant portion of their locomotor time walking long distances within their home range ([41], see also [42], [43]). This follows other postural adaptations of effective forelimb elongation in baboons, such as adopting digitigrade hand postures when walking [33], [44]–[46]. In fact, baboons start to habitually use a digitigrade hand posture early in their development, possibly as soon as two months of age [47]. Thus, by adopting extended elbow joints very early in ontogeny, baboons may already be approaching the limits to joint ranges of motion in the forelimb and thus cannot extend their forelimbs any more during maturation.

Why then are the hind limbs also not as extended early in ontogeny in baboons, and possibly other cercopithecoid monkeys? Increasing effective limb length should be just as beneficial in the hind limb as in the forelimb. One possibility may be that younger baboons need to maintain crouched hind limbs to increase maneuverability (e.g. [48], [49]); the hind limbs are used more in propulsion than are the forelimbs [32]. Greater maneuverability and agility is important for younger animals for several reasons. For example, younger baboons may need to be more agile to be able to follow their mothers closely during group dispersal events (i.e., being able to catch up to group leaders), to better avoid terrestrial predators [50], or because of social dynamics (i.e., submissive displays to older individuals, play behavior). However, as noted above, crouched limbs come with the tradeoff of an increase in musculoskeletal stresses. This may be a reason why younger animals tend to have relatively stronger long bones than older animals. In both precocial (rodents, lagomorphs, bovids) and altricial (primates) mammals, bending strength and geometric safety factors scale with negative allometry as animals grow in body size and develop with age [51]–[54]. In baboons specifically, Ruff [55] found that younger individuals of a closely related baboon subspecies (*Papio hamadryas cyncocephalus*) have relatively stronger femora than adults. Ruff [55], citing published bone biology literature, originally suggested that the overall age-related decline in relative femoral strength could be compensated by an increase in bone mineral strength. But, it is also likely that habitually adopting a more extended hind limb at older ages and when masses are greater could also attenuate musculoskeletal stresses in their relatively weaker femora. Interestingly, while the baboon femur decreases in bone strength during ontogeny, Ruff [55] also documented that the strength of the humerus actually appears to decrease at a faster rate than femoral strength, especially after 2.5 years in age. This discrepancy in humerus to femur bone strength later in ontogeny may further explain why baboons always have forelimbs that are more extended than their hind limbs (Table 1; see also [30]) and why the elbow joint remains extended over ontogeny (see above).

Another possibility, although not mutually exclusive with those proposed above, may be related to different allometries of distal limb-segment growth. In building upon Biewener's biomechanical model, Polk [9] proposed that among animals of similar body size, those with longer distal limb segments will adopt more extended postures in order to compensate for longer moment arms at the knee or elbow joint (see Figure 1 in [9]). In baboons (*Papio hamadryas cyncocephalus*), Raichlen [56] documented that the relative length of the leg segment (tibia/fibula) significantly increases over ontogeny whereas the relative length of the forearm segment (ulna/radius) does not; thus, older individuals have relatively longer distal hind-limb versus distal forelimb segments. Older baboons may then attenuate relatively larger knee joint moments by adopting an extended posture, but may not be necessary in the forelimb since the forearm does not increase in relative length, thus accounting for the fore- vs. hind limb scaling difference observed in this study.

The observed relationship between knee angle and body mass is similar when all animals are included in the analysis, and when males are examined independently. In contrast, we found no significant relationship between any of the limb posture variables and mass in the females. Behavioral differences between males and females may contribute to the observed differences in scaling relationships. Adopting more erect limb postures may come with the cost of decreasing agility, acceleration and overall speed [48], [49]. In general, these costs may be detrimental for habitually terrestrial primates like baboons when trying to escape from predators. For females this loss would additionally hamper their ability to compete with other females of the same group for resources [57], as well as to avoid aggression from dominant males in the group. Their smaller body sizes (on average of 15 kg) relative to males also reduce their ability to resist attack during bouts of male-male competition [58]. While these are all possibilities, results of the ANCOVAs comparing males and females demonstrated no statistically significant sex differences in knee angle after controlling for body mass (Table 3). One possible explanation for this result could be related to the fact that our sample of females is smaller than males (13 vs. 20 individuals, respectively), and the females encompassed a smaller range of sizes and ages (Table 1). Thus, the data from females in this study does not span a full ontogenetic spectrum, especially at younger ages. The potential for sex-based differences in joint angles remain intriguing, however, as such effects may be indicative of potential tradeoffs between locomotor biomechanics and social dynamics. Larger data sets with known dominance ranks, for example, would be necessary to fully investigate this hypothesis (e.g. [59]).

Biewener's [4], [5] biomechanical model hypothesizes that postural accommodations by anti-gravity muscles are most constrained in larger animals because small taxa (0.01–1.0 kg) can operate at higher bone safety factors relative to their body size. Recently, Polk and colleagues [10], investigated whether smaller primate taxa are indeed less constrained in adopting a specific hind limb joint orientation by examining variability in knee angles. Their results indicate that smaller taxa display more variation in knee joint angles when compared to larger species; and the latter consistently had more extended knees. In our intraspecific, ontogenetic sample of chacma baboons, we observed significantly higher levels of variation (as identified by the CV around mean values) in knee joint angles in smaller, younger individuals. Again, no relationship was found for the elbow joint. Thus, the results reported here further support Biewener's [4], [5] biomechanical model and support Polk et al. 's [10] conclusions of larger primates being more biomechanically constrained in their choice of hind limb posture.

## Conclusions

Though large, interspecific data are conducive for investigating emergent, large-scale properties among species [4], investigation of these patterns within clades [12], or within species (this study; see also [19], [22]), often deviate from the larger scale patterns in biologically meaningful ways. In chacma baboons, we found that Biewener 's [4] biomechanical model of size-dependent limb posture was supported across a broad range of body sizes for the knee joint but not the elbow joint. Furthermore, we found additional evidence that limb posture variation is size dependent for the knee joint, but again, not for the elbow. While the lack of congruence between the fore- and hind limbs may be specific to chacma baboons, it is also likely that other large-bodied terrestrial primates may exhibit similar patterns. This possibility, however, can only be fully tested with additional data. Like others (e.g. [60]), we also propose that using field data from wild animals holds promise for future studies of comparative biomechanics, as it permits data collection from a larger, more diverse population of naturally behaving individuals. Primates are an especially amenable group for field-collected data as they can be acclimated to human contact and observation, and large populations of semi-free ranging animals are also available at primate-centered biomedical facilities [61], [62]. Our results underscore how field data can provide a wealth of opportunity to address large-scale biomechanical questions not possible in animal gait laboratories. By collecting these data in the field, future studies will be able to tackle previously unaddressable questions related to sexual dimorphism, ontogeny patterns, and effects of social structure (e.g., dominance and rank) on posture and locomotion.
